# Display of Bacterial Exochitanase on *Bacillus subtilis* Spores Improved Enzyme Stability and Recyclability

**DOI:** 10.3390/molecules29184302

**Published:** 2024-09-11

**Authors:** Mati Ullah, Yutong Xia, Dalal Sulaiman Alshaya, Jianda Han, Kotb A. Attia, Tawaf Ali Shah, Huayou Chen

**Affiliations:** 1 School of Life Sciences, Jiangsu University, Zhenjiang 212013, China; matiesh@ujs.edu.cn (M.U.); sadaktk@gmail.com (Y.X.); 2222217014@stmail.ujs.edu.cn (J.H.); 2 Department of Biology, College of Science, Princess Nourah bint Abdulrahman University, P.O. Box 84428, Riyadh 11671, Saudi Arabia; dsalshaya@pnu.edu.sa; 3 Center of Excellence in Biotechnology Research, King Saud University, P.O. Box 2455, Riyadh 11451, Saudi Arabia; kattia1.c@ksu.edu.sa; 4 College of Agriculture Engineering and Food Science, Shandong University of Technology, Zibo 255000, China; tawafbiotech@yahoo.com

**Keywords:** bacterial exochitinase, *Bacillus substilis* WB800N, spore surface display

## Abstract

Chitin is the second most prevalent polysaccharide found in nature, following cellulose. Amino-oligosaccharides, the byproducts of chitin degradation, exhibit favorable biological properties and potential for various uses. Chitinases play a crucial function in the breakdown of chitin, and their exceptionally effective production has garnered significant interest. Here, in this study, the exochitinase PbChiA, obtained from *Paenibacillus barengoltzii*, was recombinantly produced and immobilized using the CotG surface protein of *Bacillus subtilis* WB800N. The resulting strain *Bacillus subtilis* WB800N pHS-CotG-Chi exhibited exceptional heat stability and efficacy across various pH levels. The chitinolytic activity of the enzyme, which had been isolated and immobilized on the spore surface, was measured to be approximately 16.06 U/mL. Including Ni^2+^, Zn^+2^, and K^+^, and EDTA at various concentration levels in the reaction system, has significantly enhanced the activity of the immobilized enzyme. The immobilized exochitinase demonstrated a notable rate of recycling, as the recombinant spores sustained a relative enzyme activity of more than 70% after three cycles and 62.7% after four cycles. These findings established a basis for additional investigation into the role and practical use of the immobilized bacterial exochitinase in industry.

## 1. Introduction

In living organisms, chitin is the second most common biopolymer. It makes up the majority of the exoskeleton of arthropods and the cell wall of fungi. Chitin is ranked second among the globally most abundant biopolymer resources. This alkaline polysaccharide is primarily extracted from the shells of marine crustaceans [1]. Chitin is extensively utilized in medicinal products due to its wound-healing capabilities, as well as in the manufacturing of industrial fabrics and feed supplements [2,3,4]. The process of hydrolyzing chitin results in the production of N-acetylglucosamine (GlcNAc) and Diacetylchitobiose (GlcNAc)2. These compounds have immunostimulatory, antioxidant, and antibacterial characteristics, as reported earlier [5,6,7,8].

The chitinase enzyme class represents those glycosyl hydrolases which hydrolyze the β-1,4-glycosidic bonds of chitin in chitin degradation systems, which yield chitin oligosaccharide or monosaccharide. Predominantly, chitinases are categorized under the glycosyl hydrolase (GH) 18 and 19 families, while a few can be found in the GH 20, 23, and 48 families [9]. GH18 family chitinases are widely found in various organisms and possess an impressive capacity to break down chitin [10]. Chitinolytic enzymes fall into two main groups, known as endochitinases and exochitinases, according to how they break down chitin chains. Endochitinases (EC 3.2.1.14) non-specifically cleave the β-1,4-glycosidic linkages in chitin and produce N-acetyl chitooligosaccharides (N-acetyl COSs). Exochitinases (EC 3.2.1.52) are further divided into two groups called chitobiosidases (EC 3.2.1.29) and N-acetyl β-1,4-D-glucosaminidases (EC 3.2.1.30). Chitobiosidases release diacetylchitobiose units in a cascading manner as the sole product from chitin, while N-acetyl β-1,4-D-glucosaminidases break down diacetyl chitobioses and N-acetyl COSs [11].

Endospores, or spores, are dormant cellular structures formed by numerous bacterial species as a response to unfavorable growth conditions [12]. These spore-forming bacterial species are able to resist quite undesirable conditions that include nutrients and deprivation, sharp fluctuations of temperature and pH, ultraviolet irradiation, and toxic substances. [13]. Though metabolically inactive, the spore demonstrates the ability to sense its surroundings and react to environmental cues conducive to cell proliferation by initiating germination and producing a new vegetative cell [14]. The unique structural characteristics of the spore contribute significantly to its germination and resilience, a subject extensively investigated in *Bacillus subtilis*, renowned as the primary model organism for spore-forming bacteria [13,15]. Spores of *B. subtilis* are composed of a partially dehydrated cytoplasm (core) encased in multiple protective layers, including the multilayered, proteinaceous coat, the thick peptidoglycan-like cortex, and the crust, which is the outermost layer composed of proteins and glycans. The exosporium, a protective shell primarily composed of glycoproteins, is the outermost layer of the coat in certain species, such as *Bacillus anthracis*, *Bacillus cereus*, and *Bacillus megaterium* [15].

Recently, the *Bacillus substilis* spore surface display system has gained interest in enzyme immobilization [16,17]. The surface display of *Bacillus subtilis* spores reduces the impact of other proteins on the displayed enzyme proteins and makes them stable and recyclable. This merit of the spore display system could make the industrial application of chitinase a reality. In addition, spores of *B. subtilis* have evolved into an excellent immobilized carrier, whose use is quite extensive due to its simple handling, shorter application time, natural resistance, stability, and recyclability in industrial applications that include foods, feeds, medicine, vaccines, antibodies, and peptide preparation [16,18].

There are several coat proteins in *Bacillus subtilis* used as an anchor protein. These include CotX, CotZ, CotY, CotA, CotB, and CotG. These anchor proteins generate fusion proteins with a target protein. The CotG-like protein family exhibits conservation among spore formers belonging to the *Bacillus* genus. In *Bacillus subtilis*, CotG exists in two forms within sporulating cells. One form is a phosphorylated protein that assembles around the developing spore. The other form is an unphosphorylated protein that is likely unfolded and accumulates in the cytoplasm of the mother cell. In the mother cell cytoplasm, unphosphorylated CotG forms aggregates that capture other coat proteins, including CotU and CotC [19].

Enzyme immobilization techniques have been proven to be one of the most effective methods for enzyme preparations, enhancing the stability of the enzyme, facilitating commercial availability, and increasing economic value. Because *Bacillus subtilis* has very simple nutritional requirements and can efficiently secrete large amounts of proteins and other metabolites, it is widely used as a safe food supplement in animal feed. Due to its production of spores that show outstanding resistance, it can be easily stored and hence is a good candidate for the fermentation industry as a carrier for bioactive molecules [20].

In the present work, we have expressed and characterized the bacterial exochitinase using a *Bacillus substilis* spore surface display system. This is the first report of using a *Bacillus subtilis* surface display system for the expression and characterization of bacterial exochitinase.

## 2. Results

### 2.1. Cloning, Transformation, and Construction of Fusion Protein

**Figure 1:**
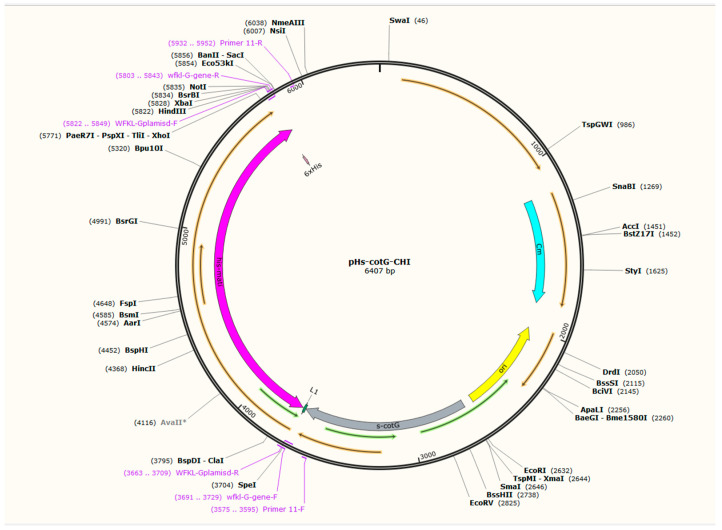
The constructed pHS-CotG recombinant plasmid map.

The recombinant pHS-CotG plasmid map is shown in Figure 1. The exochitinase gene sequence was amplified using specific primers and gel purified. The purified gel products were double digested using SpelI and HindIII restriction enzymes. Similarly, the plasmid pHS-CotG was double digested using the same enzymes. The digested gene and plasmid were ligated using T4 ligase. The ligated products were transferred to competent *E. coli* DH5α cells. The positive clones with inserted ligated products were confirmed by both colony PCR and Sanger sequencing. The pHS-CotG plasmid containing the exochitinase gene was isolated and transferred to *Bacillus subtilis* WB800N using the heat shock method and the transformation was confirmed by colony PCR and sangar sequencing prior to protein induction.

### 2.2. SDS-PAGE and Western Blot Confirmation of Displayed Exochitinase

**Figure 2:**
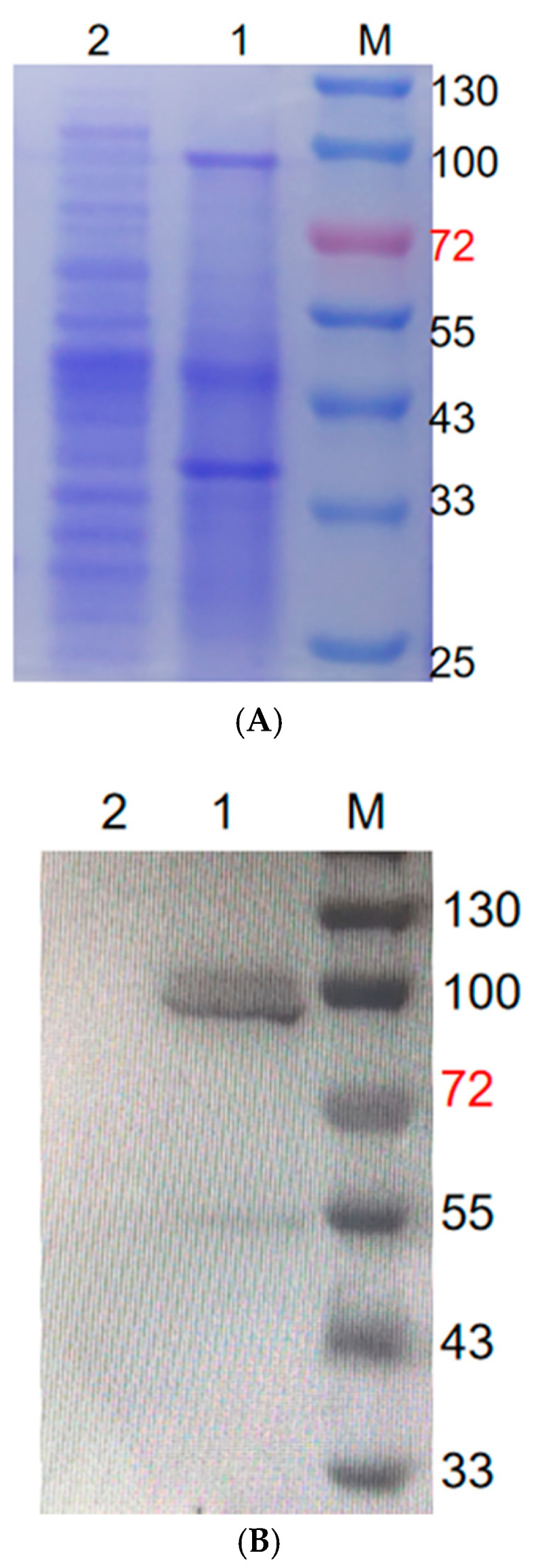
SDS PAGE and Western Blot validation of displayed protein. (**A**) shows SDS-PAGE validation of spore surface-displayed protein. M represents marker; lane 1 represents enzyme expression pHS-CotG-Chi, and lane 2 represents pHS-CotG (control). (**B**) shows the Western blot validation of spore surface-displayed protein. M represents the protein Marker; lane 1 represents protein expression by pHS-CotG-Chi, while lane 2 represents control pHS-CotG. Size of marker and proteins are indicated in kDa.

The expression of the desired exochitinase protein by the immobilized spore was confirmed by SDS-PAGE and Western blot analysis, as shown in Figure 2A,B. The recombinant *Bacillus subtilis* WB800N strains containing the pHS-CotG-Chi plasmid and the one with the pHS-CotG plasmid (no exochitinase gene) were inoculated into DSM media containing chloramphenicol and kanamycin antibiotics for 36 h for protein expression. After the extraction of spore surface proteins, the CotG-Chi fusion protein (95.9 kDa) was confirmed by SDS page and Western blot, as shown in Figure 2A,B. The lane 1 in Figure 2A,B represents the expression and confirmation of the exochitionase gene.

### 2.3. Immunofluorescence Assay of Immobilized Exochitinase

**Figure 3:**
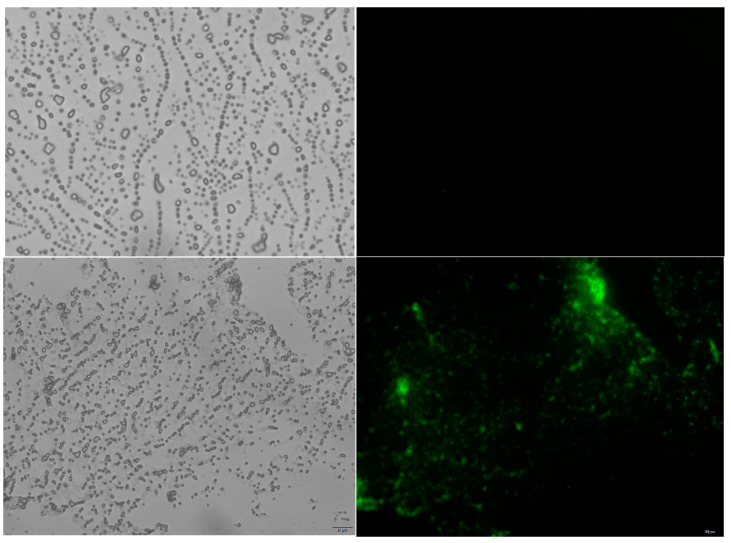
The immunofluorescence and bright-field microscopy examination of the immobilized recombinant protein CotG-Chi. The top images represent CotG, while the bottom images represent CotG-Chi.

The recombinant exochitinase display on the spore surface of *Bacillus subtilis* WB800N was investigated by an immunofluorescence technique, as shown in Figure 3. Both the fusion protein and coat protein, that were tagged to the recombinant exochitinase, were recovered from the spore surface of the *B. subtilis* WB800N pHS-CotG and *B. subtilis* WB800 pHS-CotG-Chi, respectively. Briefly, the culture media containing *B. subtilis* spores were centrifuged at 8000 rpm for 15 min. Next, 10 mL Buffer GTE containing lysozyme (20 mg/mL) were added to the pelleted spores and incubated at 37 °C for one hour. Following this, the spores were washed twice with PBS buffer and stored until further use. As shown in Figure 3, the fusion protein that contains exochitinase showed visible green fluorescence, whereas the control *B. subtilis* WB800N pHS-CotG surface, which displayed the coat protein, revealed no detectable fluorescence. This clearly suggests the successful display of the recombinant exochitinase on the spore surface of *Bacillus subtilis* WB800N.

### 2.4. Enzyme Activity Assay of Displayed Protein

**Figure 4:**
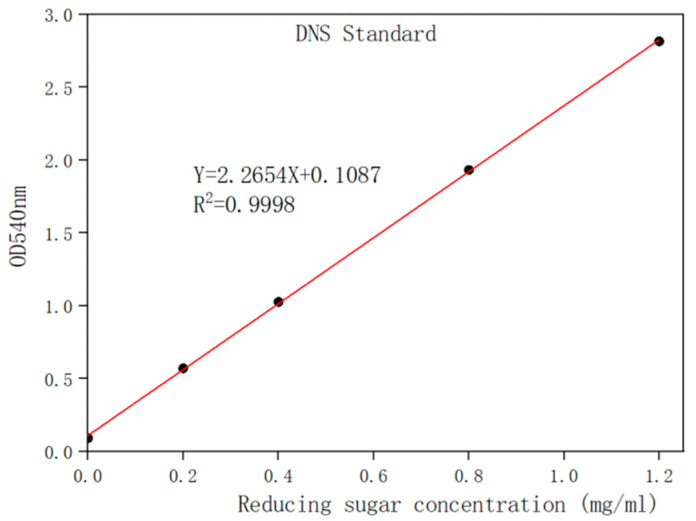
The standard curve of the reducing sugar concentration determined by the DNS method.

The exochitinase activity of the spore was determined by a 3,5-dinitrosalicylic acid (DNS) colorimetric method. The standard for the enzyme activity is shown in Figure 4. For the measurement of chitinase activity, 100 ul of spore was properly mixed with 400 ul of buffer (50 mM of phosphate buffer) and 500 ul colloidal chitin. The reaction mixture was kept at 50 °C for 1 h. Further, the reaction mixture was centrifuged and supernatant was collected and mixed with 1 mL of DNS reagent. After the addition of DNS, the solution was kept in boiled water for five minutes followed by immediate cooling to room temperature. Finally, the absorptive value was measured at OD540 nm. The enzyme activity of the spore was defined as the amount of enzyme required to release 1 μ mol of reduced sugar per minute under this condition, using GlcNAc as the standard. The activity of the spore was noted as 16.06 U/mL.

### 2.5. The Effect of Temperature and pH on the Activity and Stability of the Immobilized Enzyme

**Figure 5:**
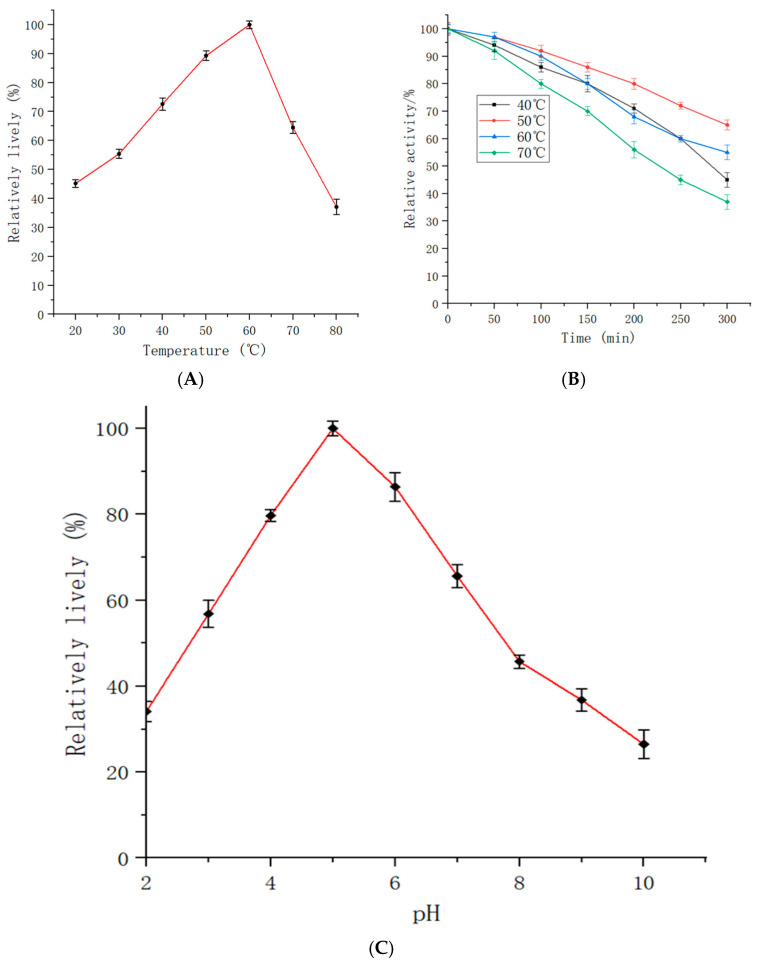
The effect of temperature and pH on enzyme activity. (**A**) represents the effect of temperature, (**B**) represents the thermal stability of exochititnase at different temperatures, while (**C**) represents the best pH and relative activity of chitinase at different pHs.

Both temperature and pH are important factors in the catalytic reaction and degradation of chitin by chitinase. The CotG-Chi displayed on the *Bacillus subtilis* WB800N spore showed its enzymatic activity within a broad pH range, of which pH 5.0 was found to be the best value (Figure 5C), showing the enzyme adapting to acidic conditions. The pH stability was checked and the analysis has revealed that, at pH 4.0–6.0, the residual enzyme activity of the enzyme was maintained above 80%, as shown in Figure 5C. The best temperature of the CotG-Chi displayed on *Bacillus subtilis* WB800N was 60 °C. The enzyme stability was observed as high at the temperature range of 40 °C to 60 °C, with activity above 70%. However, when the temperature exceeds 65 °C, the enzyme activity was noted as declining, as shown in Figure 5A. Further, the immobilized Chi was analyzed for thermal stability at different temperatures and time durations. The solutions of the reaction system including the enzyme were maintained at 40 °C, 50 °C, 60 °C, and 70 °C for different periods of time. In accordance with the measurement of the enzyme activity, the temperature tolerance was noted. As shown in Figure 5B, under the conditions of 50 °C for 3 h, more than 80% of the enzyme activity was retained. Similarly, at a temperature range of 40 °C to 70 °C, the residual enzyme activity still reached more than 70% after two and a half hours. As shown in Figure 5B, residual enzyme activity after 5 h was found to be above 50% at the temperatures of 40 °C, 50 °C, and 60 °C, respectively. This thus indicates the effective preservation of the viability and thermal stability of immobilized exochitinase.

### 2.6. The Effect of Chemical Substances and Metal Ions on Enzyme Activity

**Figure 6:**
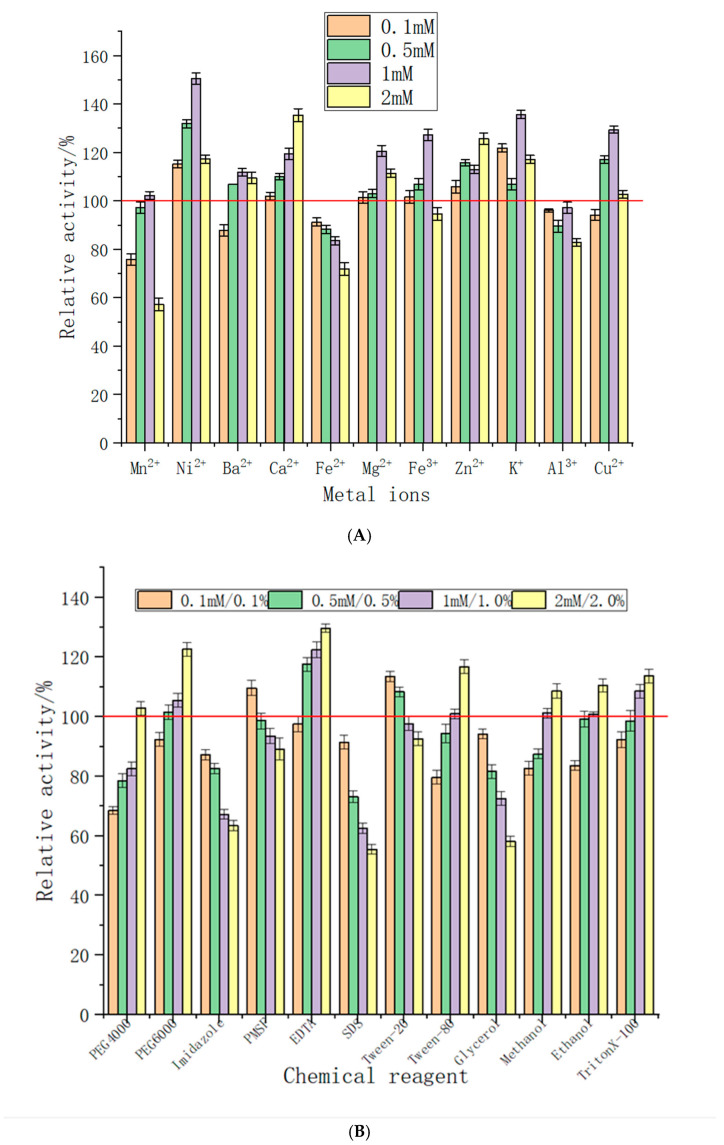
(**A**) The effect of different concentrations of metal ions and chemical reagents on chitinase activity; (**B**) the effect of different concentrations of chemical reagents on chitinase activity.

To investigate the effect of chemical substances and metal ions on immobilized enzyme activity, a range of different ions and chemical substances at different concentrations were analyzed. The supportive and inhibitory effects of different metal ions are shown in Figure 6A. The results showed that Mn^2+^, Fe^2+^, and Al^3+^ had different inhibitory effects on surface-displayed immobilized chitinase, as shown in Figure 6A. With the increase in concentration, the inhibitory effects of Fe^2+^ on chitinase became stronger and the activity of the enzyme was lowered to 70% of the original level. However, Ni^2+^, Zn^+2^, and K^+^ has increased the surface-displayed immobilized enzyme activity at different concentration levels. The enzyme activity after the addition of Ni^2+^ increased for all molar concentrations, with highest value of 0.5 mM reaching approximately 150% of the original enzyme activity. Similarly, the different molar concentrations of Zn^+^ and K^+^ increased the enzyme activity to approximately 120% and 140%, respectively (Figure 6A). The remaining ions have intermediate inhibitory and supportive effects on the surface-displayed immobilized enzyme activity, as shown in Figure 6A.

Several compounds, including SDS, imidazole, and glycerol have been found to limit chitinase activity, as shown in Figure 6B. The inhibitory effect of these chemical compounds became stronger as the molar concentration in the reaction system increased. While the majority of chemical substances were observed to inhibit or decrease the immobilized enzyme activity, higher concentrations of EDTA and Triton X-100 had a stimulating effect. In particular, EDTA significantly increased the activity of the immobilized enzyme by approximately 127%, as depicted in Figure 6B.

### 2.7. Recovery Rate of Immobilized Exochitinase

**Figure 7:**
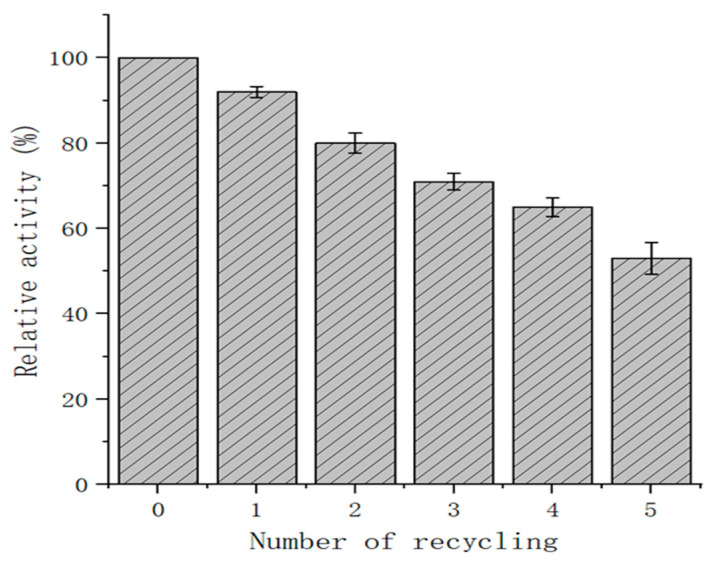
Relative Activity of immobilized recovered exochitinase after different recycling cycles.

The immobilized exochitinase recycling rate was determined by the centrifugation of the reaction solution in the first reaction and the washing with PBS of the recovered budding spores for the next reaction, which was repeated five times. As shown in Figure 7, the recombinant spores were found to have a relative enzyme activity of over 70% after three cycles. In addition, 62.7% of the residual enzyme activity was retained after the fourth cycle of recycling. This therefore proves that an enzyme in its immobilized form has an enormously high recyclability compared to being in its free form. It is also much more convenient and time-saving to use, which highlights the advantages in using immobilized enzymes through the coat surface display system of *Bacillus subtilis*.

## 3. Discussion

Chitinases have garnered significant interest recently because of their considerable potential in industrial applications [21,22]. Up to now, many microbial endochitinases have been explored and characterized using *E. coli* as an expression host [23,24]. On the other hand, there is no report on the expression of exochitinases utilizing *Bacillus substilis* spore surface display systems. Herein, we employed a *Bacillus substilis* spore surface display expression system for the heterologous expression and characterization of an exochitinase from *Paenibacillus barengoltzii*. More specifically, the exochitinase gene from *Paenibacillus barengoltzii* was genetically displayed on the *B. subtilis* spore surface by using the coat protein CotG. Prior studies have utilized this particular coat protein as a means of displaying different enzymes [25,26].

The current research on the *Bacillus subtilis* spore coat-displayed exochitinase system can be of immense importance and perhaps has its potential application as a biopesticide in the agricultural industry. Chitinase inhibits the growth of many pathogenic fungi, which is considered a serious concern to worldwide agriculture. Rostami et al. expressed chitinase on the surface of *B. subtilis* spores as a biological control agent. This enzyme has been demonstrated to efficiently inhibit the growth of the fungi *Rhizoctonia solani* and *Trichoderma harzianum* in in vitro experiments [27]. Moreover, this spore coat-displayed exochitinase system can be used for the delivery of the therapeutic exochitinase to targeted sites such as infected wounds or areas with chitinous biofilms, and for the production of chitosan from chitin for industrial applications.

Several coat proteins act as an anchor protein in *Bacillus subtilis*. Earlier, CotB was used as the anchor protein, mainly for the display of the tetanus toxin on the outer layer of *B. subtilis* spores [28]. Similarly, for the successful display of the green fluorescent protein, CotY was used as a molecular carrier [29]. CotZ was used to show the successful display of large extrinsic proteins [25]. CotG has been used consistently as a carrier protein for the display of highly active enzymes on the spore surface [25,26]. In the case of the *B. subtilis* spore surface display, a number of carrier proteins that could be exploited were identified. Only a few of these anchoring proteins turned out to be capable of displaying the enzyme on the spore surface and retaining their enzymatic activity. The anchor protein used in the present study was the coat protein CotG. For the construction of a fusion protein, exochitinase fusion was shown on the spore surface of *B. subtilis* as an immobilization of exochitinase.

Metal ions and chemical reagents have different supportive and inhibitory effects on the activity of immobilized enzymes, as shown in Figure 6A,B. Most of the chemical reagents have inhibitory effects on the immobilized enzyme activity, with few exceptions, the most notable of which was EDTA, which has obviously improved enzyme activity at different molar concentrations (Figure 6B). In a previous study, EDTA was found to decrease the activity by 38.7%, which contradicts our analysis; however, SDS and ethanol deactivated the enzyme, which coincides with our findings [30]. Similarly, several other studies have reported the inhibitory effect of SDS on chitinase activity [31,32]. Many studies have confirmed the role of inorganic salts in spore formation. Added to a medium, inorganic salts, such as those with high concentrations of Mg^2+^, K^+^, and Ca^2+,^, will have an impact on the formation of spores [33]. Previously, Na^+^, K^+^, Mg^2+^, and Ca^2+^ were shown to improve the stability and activity of chitinase from a novel gram variable *Bacillus* species [30]. Chitinase activation by Ca^2+^, Mg^2+^, Mn^2+^, Na^+^, and K^+^ was also reported by many authors [30,32,34,35]. In contrast, Ag^2+^, Hg^2+^, Fe^2+^, Zn^2+^, Cu^2+^, and Zn^2+^ were found to play a role in the inhibition of chitinase activity [36,37]. Yet, few ions, such as Cu^2+^ and Zn^2+^, could act as chitinase stimulators times [31,38], although at others could be chitinase inhibitors [37,39]. In our analysis, Mg^2+^, K^+^, and Ca^2+^ at different molar concentrations were found to increase the activity of the immobilized enzyme (Figure 6A). Metal ions play a role in stabilizing the molecular structures of proteins and in the catalytic activity of numerous enzymes. Heavy-metal ions have a tendency to regularly cause the denaturation of enzyme proteins.

The recycling rate of the enzyme is of great importance related to the commercial application of enzyme preparation [40]. The simple washing of spores is one of the attractive characteristics for industrial application. Herein, through three rounds of recycling, the residual enzyme activity of the exochitinase was maintained above 70%. This demonstrates the higher efficiency of exochitinase recyclability than that of cellulase immobilized on polyvinyl alcohol/Fe_2_O_3_ [41]. As such, this constitutes a simple and feasible method of displaying bacterial exochitinase as an immobilized enzyme, providing a way for its large-scale commercial production and application.

The *Bacillus subtilis* spore surface display technique helps to immobilize and heterologously express industrial enzymes. However, its use with the exochitinase enzyme, a chitin hydrolase, has limits. This technology requires fine-tuning genetic and metabiological functions for efficient display. For long-term storage and use, the protein on the spores must be stable. Last but not least, protein presentation might be difficult to sustain from the lab to industrial scale. Due to these limitations, researchers are exploring genetic engineering, protein engineering, and fermentation process improvements to optimize *Bacillus subtilis* spore surface display system technology to improve protein expression, stability, and application potential. Research and technological innovation should overcome these restrictions and make *Bacillus subtilis* spore surface display system technology more important in production.

## 4. Materials and Methods

### 4.1. Purification of Chi Protein and Preparation of Polyclonal Antibody

First, the exochitinase gene of *Paenibacillus barengoltzii* was expressed in the *E. coli* BL21 strain. The expression of this target gene was checked by the *E. coli* pET-21a-Chi recombinant engineering strain. Pure Chi protein with bioactivity was obtained after inclusion body washing and renaturation, followed by Ni–NTA affinity chromatography and purification. The purified Chi protein was used to immunize New Zealand white rabbits using AmiconUltra-15 ultrafiltration tubes, MilChiore, MWCO10 KD (Merck KGaA, Darmstadt, Germany). Using the Chi protein as the antigen, the polyclonal antibody was prepared through multiple injections: 300 mg Chi + Freund complete adjuvant in the first week and 100 mg Chi + Freund incomplete adjuvant every 2 weeks thereafter. To be more specific, Western blotting was performed to assess the titer of the polyclonal antibody. The primary antibody was an antiserum from New Zealand white rabbits that were immunized, while the secondary antibody was Affinipure HRP conjugate goat anti-rabbit IgG. The antiserum was diluted 100 times, 200 times, 400 times, and 600 times in TBST buffer, while the secondary antibody was added at a ratio of 1:7500. Color development was finally carried out using the Omni-ECLTM Basic chemiluminescence kit, with its detection conducted on a Chemiluminescence Imager.

### 4.2. Construction of Recombinant Plasmids and Transformation

Using the Vazyme genome extraction kit, the genomic DNA of *B. subtilis* WB800N was extracted. The CotG gene fragment of capsid protein was amplified using primers CotG-F and CotG-R that introduced the flexible linker peptide composed of GGGGS at the C-terminus of CotG. The CotG gene fragment was inserted into the pHS plasmid by Hind III and Spe I restriction endonucleases to construct the pHS-CotG recombinant plasmid. The plasmid map is shown in Figure 1. The Chi gene fragments were amplified by Chi-F (Spe I) and Chi-R (Hind III) and inserted into the pHS-CotG plasmid using Spe I and Hind III restriction enzymes, which created the recombinant expression vector pHS-CotG-Chi. The plasmids pHS-CotG and pHS-CotG-Chi were transformed into *E. coli* DH5α. Further, plasmid transformation to the *E. coli* was confirmed after growth and colony PCR and was processed for plasmid isolation. The extracted recombinant plasmids from *E. coli* were transformed into *B. subtilis* WB800N competent cells to construct *B. subtilis* WB800N pHS-CotG and *B. subtilis* WB800N pHS-CotG-Chi recombinant bacteria. The summary of the plasmids, strains, and primer sequences is listed in Table 1.

### 4.3. Spore Preparations and Processing

The WB800N bacterial strain of recombinant *B. subtilis* was cultured at 37 °C in an LB medium supplemented with chloramphenicol and kanamycin antibiotics at 180 rpm for 12 h. After that, 1% inoculum volume was transferred to a DSM liquid medium with the same antibiotics and incubated at 37 °C for 36 h to induce spore development. The bacterial pellet was spun down, and was lysed by the GTE buffer with lysozymes at 37 °C for one hour. Lastly, the lysed spores were centrifuged and washed with the PBS buffer.

### 4.4. SDS-PAGE and Western Blot Analysis of Recombinant Exochitinase

The *Bacillus subtilis* spores were subjected to centrifugation and then re-suspended in a decoating extraction solution of the same volume. Afterwards, they were exposed to a temperature of 70 °C for a period of one hour. Consequently, the fusion protein CotG-Chi, which was being displayed on the spore surface, was detached from the spores. Then, SDS-PAGE and Western blotting analysis verified the extracted proteins. The primary antibody used was rabbit anti-Chi, at a 1:100 dilution. The secondary antibody used was the HRP-conjugated Affinipure Goat Anti-Rabbit IgG, at 1:10,000, developed in the Omni-ECLTM Basic Chemiluminescence Kit from Epizyme Biotech.

### 4.5. Examination of Bacterial Spore Using Immunofluorometric Method

A 10 µL volume of the appropriately diluted spore suspension was placed on a slide that had been pre-soaked in 0.1% *w*/*v* gelatin. The slides were allowed to dry at room temperature for 1–2 h and then washed three times with PBS buffer. Next, the slides were immersed in PBS buffer containing 5% skimmed milk powder for 30 min. The samples were further incubated overnight at 4 °C using rabbit anti-Chi serum at a dilution of 1:100. Following this step, the slides were washed three times with PBS solution. Subsequently, the slides were incubated overnight at 4 °C with antirabbit IgG-FITC conjugates containing fluorescein isothiocyanate at a dilution of 1:200. Finally, the slides were rinsed with PBS buffer and examined under an inverted fluorescence microscope.

### 4.6. Enzyme Activity Assay of Displayed Protein

The immobilized chitinase activity was evaluated based on the description provided by Lee et al. [42], with some minor modifications. The reaction mixture consisted of 100 ul of appropriately diluted spore, 400 ul of PBS solution, and 500 ul of a 1% (*w*/*v*) colloidal chitin solution. The mixture was thoroughly mixed and incubated at 50 °C for one hour. The amount of released reducing sugars was then determined using the dinitrosalicylic acid (DNS) technique [43]. The definition of one unit (U) of chitinase activity is the quantity of enzyme needed to liberate 1 μmol of reducing sugars per minute, with N-acetyl glucosamine as the reference standard, under the specified conditions.

### 4.7. The Effect of pH and Temperature on the Activity and Stability of Immobilized Enzyme

In order to investigate the effects of temperature and pH on the immobilized recombinant exochitinase enzyme displayed on the surface of *Bacillus subtilis*, the enzymatic reaction system used different temperatures and pH levels. The pH range of the reaction setup was adjusted from 2.0 to 10.0, with increments of 1 unit, at a temperature of 50 °C. The activity of the Chi enzyme under different pH conditions was observed to determine the optimal pH for the reaction. The relative enzyme activity was calculated by setting the peak activity at 100%, allowing us to identify the optimal pH. Next, we set the buffer pH at 5.0 and varied the temperature of the reaction system from 20 to 80 °C, with increments of 10 °C. We observed the enzyme activity at different temperature settings to determine the most suitable temperature for the reaction system. The changes in enzyme activity corresponding to the temperature variations were recorded for analysis.

The determination of temperature tolerance involved adding a spore suspension to the buffer and initiating the reaction by adding colloidal chitin at four different temperature gradients: 40 °C, 50 °C, 60 °C, and 70 °C. The reaction was allowed to proceed for specific time intervals of 0 min, 50 min, 100 min, 120 min, 150 min, 200 min, 250 min, and 300 min. The baseline enzyme activity at 0 min was set at 100%. The enzyme activities of the experimental groups at different time points were individually assessed and computed to evaluate the thermal stability of the immobilized exochitinase.

### 4.8. Effect of Chemical Substances and Metal Ions on Enzyme Activity

To investigate the effect of different chemical substances and metal ions on the immobilized enzyme, chitinase activity was assayed at the optimum reaction temperature and optimum pH. The metals ions and chemical substances were included in the reaction system and the enzyme activity was assessed accordingly. The enzyme activity was determined relative to the baseline measurement of 100% in the absence of any added chemicals within the reaction system, and subsequently an assessment was conducted to ascertain the impact of various metal ions and chemicals on the activity of immobilized exochitinase.

### 4.9. Recovery Rate of Immobilized Exochitinase

The enzymatic efficiency and recyclability of immobilized exochitinase was conducted under the conditions of optimal pH and temperature parameters. A series of 5 reaction cycles were performed accordingly. Subsequent to each reaction, the supernatant was separated through centrifugation with the resulting pelleted enzymes being reserved for the following cycle. The activity of the enzyme in the first reaction and the efficiency of the immobilized enzyme were both taken as 100%. In this respect, residual enzyme activity and the amount of enzyme were determined after each cycle of the reaction. The recovery efficiency of immobilized exochitinase was determined based on the comparative enzyme activity.

## 5. Conclusions

The expression and characterization of exochitinase gene from *Paenibacillus barengoltzii* were effectively achieved using the *Bacillus substilis* WB800N spore surface display system. The immobilized enzyme exhibited excellent pH and heat stability while catalyzing chitin breakdown activities. The impact of metal ions and chemical reagents on the immobilized enzyme activity exhibited notable variations. Specifically, Mg^2+^, K^+^, Ca^2+^, and EDTA were found to have a positive effect on the catalytic reaction. The immobilized exochitinase exhibited a significant recovery rate, with the recombinant spores maintaining a relative enzyme activity of over 70% after three cycles and 62.7% after four cycles. The simple PBS washing technique of spores and high recycling usage rate serve as a valuable benchmark for the industrial use of bacterial exochitinases.

## Figures and Tables

**Table 1:** Description of plasmids, strain, buffer, and primer sequences.

Materials	Description	References
Strains		
*E. coli* BL21 (DE3)	Type strain	Our Lab
*E. coli* DH5α	Type strain	Our Lab
*B. subtilis* WB800N	Type strain	Our Lab
Plasmids		
pET-21a-Chi	pET-21a expression vector carrying the Chi gene, Amp +	GenScript
pHS	*E. coli*-*B. subtilis* shuttle vector, Cm +	Our lab
pHS-CotG	pHS derivative carrying the capsid protein CotG gene, Cm +	This work
pHS-CotG-Chi	pHS derivative carrying the fusion CotG-Chi gene, Cm +	This work
Primer sequences		
CotG-F	TACTACAAAAAACCGCACCAC	This work
CotG-R	GTTGCTGTTCCTGTTCTGAAT	This work
Chi-F	CGGACTAGTATGGCACCAGCTGATCAAGCATATAAAGTTG	This work
Chi-R	CCCAAGCTTTCGGGCTTTGTTAGCAGCCGGATCTCAGTGGT	This work
Buffer		
PBS buffer	8 g NaCl, 0.2 g KCl, 1.42 g Na_2_HPO_4_, 0.27 g KH_2_PO_4_, pH 7.4	This work
Decoating extraction buffer	1.5% SDS, 50 mM DTT	This work
GTE buffer	10 mM EDTA, 20 mM pH 7.5 Tris-HCI, 50 Mm glucose, 2 mg/mL lysozyme	This work

## Data Availability

Data are contained within the article.
